# Embodied simulations improve memory, but which is the most effective? The crucial role of action observation and its dynamic interplay with motor imagery ability

**DOI:** 10.3389/fpsyg.2026.1889010

**Published:** 2026-07-22

**Authors:** Mara Stockner, Giuliana Mazzoni, Ivan Enrici, Francesco Ianì

**Affiliations:** 1 Department of Dynamic and Clinical Psychology, and Health Studies, Sapienza University of Rome, Rome, Italy; 2 Faculty of Psychology, University of Hull, Hull, United Kingdom; 3 Department of Philosophy and Educational Sciences, University of Turin, Turin, Italy; 4 Department of Psychology, University of Turin, Turin, Italy; 5 Centro di Logica, Linguaggio, e Cognizione, University of Turin, Turin, Italy

**Keywords:** action memory, action observation, kinesthetic imagery ability, mental simulation, motor imagery

## Abstract

Literature has shown that memory can be influenced by sensorimotor simulations at encoding as those involved in motor imagery (MI), action observation (AO), or affordances elicited by visual object presentation (OBJ). Such simulations can improve memory by creating multimodal and thus, more accessible memory traces. However, no research has yet compared all these simulations within a single study and investigated their relationship with participants’ imagery ability. The current study aimed to fill this gap by assessing the effects of different simulations on episodic memory for objects that initially were encoded as part of a sentence in which the action was ‘grasping’ (e.g., You are grasping a fork). By applying a between-subject design, 163 participants were assigned to different sensorimotor encoding conditions, and their memory was tested in a free recall task 15 min after encoding. Our results showed a main effect of AO, indicating an overall AO benefit to memory enhancement. Moreover, a three-way interaction between AO, MI and OBJ showed how the facilitating role of AO can be dampened by MI and OBJ. Finally, we found that the individual trait of kinesthetic imagery (KI) interacts with AO in recall performance, as higher levels of KI interfere with AO encoding, suggesting potential different mechanisms underlying the mental simulations involved in motor imagery and action observation.

## 1 Introduction

Over the last decades, a growing body of literature suggested that memory functions are not a modal but rather represent cognitive processes influenced by sensorimotor activations (Dijkstra and Zwaan, 2014; Ianì, 2019). Based on this theoretical conceptualization of memory, a wide range of cognitive and neuroscientific research has investigated the role of sensorimotor information in memory. Several studies investigated the effectiveness of so-called “embodied mental simulations” at encoding, that is, cognitive processes that allow to simulate visual, sensory and motor aspects of the material to be learned. For instance, recent studies show that memory performance is improved both when individuals actively imagine the visual characteristics of the items to be studied, i.e., mental imagery (Oliver et al., 2016) and when they actively simulate a physical interaction with the objects, involving sensory and motor information, i.e., motor imagery (MI) (e.g., Marre et al., 2024). Marre et al. (2021) compared different simulation strategies (visual imagery, third-person motor imagery and first-person motor imagery) with a “mental rehearsal” control condition involving low-embodiment strategies (i.e., mentally rehearsing the word with no explicit perceptual and/or motor simulation instruction). They found that all simulations were more efficient than the mental rehearsal condition and that first person motor imagery was the most effective (see also Marre et al., 2024).

The beneficial effect of sensori-motor simulation on memory is not limited to mental and motor imagery. For instance, memory for action sentences such as “Peel a banana” improves when participants simulate the action through gestures involving motor components (e.g., Serino et al., 2025). Similarly, memory enhancement is observed when individuals observe the gestures of others (see Dargue et al., 2019), as well as through action observation (AO) which has been shown to enhance episodic memory performance in several studies (Bouazzaoui et al., 2024; Villatte et al., 2023). The hypothesized underlying facilitating mechanism is the motor contribution (Ping et al., 2014; Ianì and Bucciarelli, 2018), due to the mirror system, elicited by action observation (e.g., Rizzolatti and Sinigaglia, 2016). In other words, observing actions should trigger a sort of covert motor simulation, or a “motor resonance” of the main components of the observed action (Ianì, 2021).

Other forms of sensorimotor activation that seem to have a positive effect on memory are those involved in the simple visual presentation of objects. A substantial body of literature has shown that this can automatically activate affordances, i.e., the covert motor simulation of the action that usually is performed with the objects (e.g., Tucker and Ellis, 1998) that has also been shown to play a role in memory processes (e.g., Chen et al., 2024). Daprati et al. (2022) showed that memory for objects presented as words (which only indirectly prime the sensorimotor system) was generally less accurate compared to memory for objects presented as photographs, which are more likely to directly activate the sensory-motor associated affordances (a phenomenon usually called “picture superiority effect,” Whitehouse et al., 2006).

The different types of sensorimotor simulations described above share some overlapping neural and sensorimotor features (O’Shea, 2022). However, recent experimental and theoretical literature also suggests different forms of simulation (mental imagery, motor imagery, action observation) exploit, at least partially, different cognitive processes, giving rise to important differences at the phenomenological level (e.g., Reilly et al., 2024; Stockner et al., 2025b). For instance, imagery requires more deliberate simulative processes than those involved in action observation, which appears more automatic and implicit (Ianì, 2021, 2024).

Whilst the different role of different types of sensorimotor activations have been widely investigated in other domains of cognition, as motor learning (Eaves et al., 2024; Vogt et al., 2013), there is very scarce literature specifically investigating their separate and potentially complementary roles in affecting episodic memory performance. Senkfor et al. (2002) conducted an EEG experiment in which they compared action observation, first-person perspective motor imagery, enactment, and a control condition (cost estimation of the object) in a source-memory task. The authors showed distinct ERPs at occipital sites between conditions with a visual object component (enactment and AO) and conditions without (imagery and control). The difference between visual (AO and enactment) and non-visual (imagery and control) conditions was also observed in behavioral memory tasks, in which AO and enactment conditions showed the highest level of accuracy. A more recent study by Yang et al. (2024) found that recall was worse in a mental rehearsal condition compared to all other embodied conditions (action observation, motor imagery, enactment), which did not differ from each other. Regarding the specific contributions of the visual *action* or *object* presentation, Daprati et al. (2022) found comparable results in encoding conditions consisting of either only the visual *object* presentation, or the visual *action* presentation (the action-object interaction was pantomimed without the object isolating the contribution of kinaesthetic information). However, Kafkas et al. (2024) observed better memory performance for action observation with objects compared to the mere object perception during encoding (see also Lacoste-Badie and Droulers, 2014), suggesting that action observation may have a stronger effect on memory than the sensorimotor contribution of mere object perception.

Overall, these studies suggest that it is possible that different types of mental simulations may affect memory by exploiting different mechanisms. However, there is a clear lack of systematic manipulations of the various sensorimotor contributions. The present study aims to fill this gap with an experiment in which three factors (action observation—AO, motor imagery—MI and visual object presence—OBJ) were manipulated in a single factorial between-subject design. Our dependent variable was the episodic memory of objects which were initially encoded as part of ‘grasping’ sentences (e.g., grasping a fork). Although all actions were verbally labelled as ‘grasping’, the actual motor programming for each action differed. Participants’ memory for the objects was later tested verbally in a free recall task 15 min after encoding.

We expected that the sensorimotor conditions would overall improve memory performance compared to the mental rehearsal control condition. Due to the lack of specific literature, hypotheses regarding specific interactions between AO, MI, and OBJ encoding conditions were exploratory. Based on some literature, according to what we could call the “multiple sensorimotor encoding modality” hypothesis, one could hypothesise that the joint contribution of multiple sensorimotor simulations leads to better memory than single sensorimotor modalities (for example, the beneficial role of AO + MI known from other action-related fields of cognition, e.g., Eaves et al., 2016): the more modalities present, the better the memory. On the other hand, some literature suggests neurocognitive differences between simulations involved in action observation and mental imagery (Stockner et al., 2025b), so one can also speculate that action observation in conjunction with mental imagination may lead to interference effects. However, these remain speculative hypotheses.

We also measured the role of the individual ability to create mental images (both kinaesthetic and visual imagery ability), hypothesizing an increase in memory performance in high imagery ability in the MI encoding condition (Fulford et al., 2018; Palmiero et al., 2019; however, see Marre et al., 2021 for contrasting results). Finally, we also considered sensorimotor characteristics of our stimuli (the objects involved in each sentence) and associated sensorimotor simulation elicited by sentence comprehension (present in all encoding conditions). In line with the literature in other languages, higher sensorimotor characteristics of the objects should lead to stronger activations and thus, create stronger memory traces (e.g., Pexman et al., 2013; Dymarska et al., 2023), leading to increased memory performance (e.g., Sidhu and Pexman, 2016).

## 2 Methods

### 2.1 Experimental design

A 2 (Action observation/No Action Observation) × 2 (Visual object presence/No visual object presence) × 2 (Motor Imagery/No Motor Imagery) factorial design was adopted resulting in eight experimental conditions that were administered in a between-subject design. All factors were manipulated between-subjects to avoid making participants aware of the other encoding conditions and thus, eliminating the risk of related carry-over effects. The eight conditions were as follows (in conditions involving AO or OBJ, participants observed visual stimuli corresponding to the action previously described in the sentence; in conditions involving MI, participants were instructed to mentally simulate the action previously described in the sentence):

- AO/OBJ/MI: observe action video + observe object + imagine performing action
- AO/OBJ/No MI: observe action video + observe object
- AO/No OBJ/MI: observe action video + imagine performing action
- AO/No OBJ/No MI: observe action video
- No AO/OBJ/MI: observe object + imagine performing action
- No AO/OBJ/No MI: observe object
- No AO/No OBJ/MI: imagine performing action
- No AO/No OBJ/ No MI: (mental rehearsal) repeat sentence

The “control condition” (No AO/No MI/No object) consisted in mental rehearsal (i.e., sentence repetition) of the action sentence that was initially listened to in all encoding conditions (Marre et al., 2021). In line with the literature on AO used in conjunction with MI (Vogt et al., 2013), participants were asked to carry out the various encoding conditions simultaneously. In particular, in the AO/OBJ/MI condition, participants watched a video depicting the action on the object previously described in the sentence and simultaneously imagined themselves performing it, feeling the bodily sensations they would experience while carrying out the action with the object. In contrast, in the AO/No OBJ/MI condition, participants watched a video with no object, in which only the hand mimicked grasping the object, and no reference to the object was made in the instructions.

See Materials and Procedure of a more detailed description of the methodology in the various conditions.

### 2.2 Participants

Sample size was determined using the smpsize_lmm () function of the *sjstats* R package (Lüdecke and Lüdecke, 2019). The power analysis yielded a necessary minimum sample size of 16 participants per condition when including a power of 0.90, a significance level of 0.05, eight clusters (experimental conditions) and 13 observations per cluster (number of trials). Thus, a total of 163 young adults (*M*_age_ = 24.48 years; *SD* = 4.37; 105 females, 4 preferred not declaring their sex) participated in the study. Participants were recruited from university courses and via social media and did not receive any compensation for their participation.

### 2.3 Materials

#### 2.3.1 Grasping sentences

Twenty-six objects were chosen from the Italian sensorimotor norms (Repetto et al., 2023). Objects were divided into two experimental blocks that did not differ in terms of familiarity (*t*(12) = −0.12, *p* = 0.90), concreteness (*t*(12) = −1.20, *p* = 0.25), touch scores (*t*(12) = 0.61, *p* = 0.56), vision scores (*t*(12) = 0.06, *p* = 0.95) and frequency (*t*(12) = 1.05, *p* = 0.31). Half of the participants of each group, randomly selected, were presented with one block of sentences at encoding whereas the other half with the second block. Blocks of stimuli were created, ensuring different types of grasps (based on Yu et al., 2023) for each of the 13 different objects in each experimental block.

All ‘grasping’ sentences were composed of the verb in the second person “You are grasping” (in Italian “Stai afferrando…”) and the object (e.g., “… a bottle”). The same action verb (grasping) was used to have a single action verb to control for the linguistic sensorimotor characteristics related only to the objects. Moreover, the second-person approach was adopted in line with Brunyé et al. (2009) and Ditman et al. (2010) to induce a sense of engagement (involvement of the self) also in the control condition (mental rehearsal). See Table A1 for the complete list of stimuli.

#### 2.3.2 Visual stimuli

**Figure 1:**
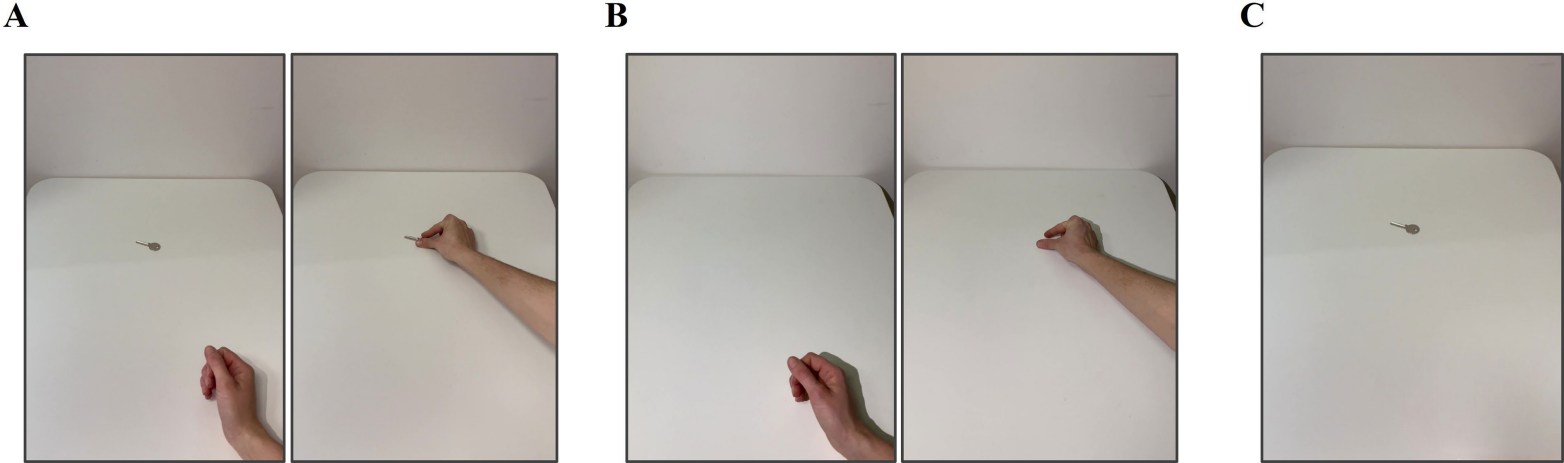
**(A)** First and last frame of a video depicting the grasping action with the object. **(B)** First and last frame of the video depicting the grasping action without the object. **(C)** Still frame used for the condition containing the object but no action.

All videos used in the conditions containing action observation lasted 3.5 s and were filmed in first-person, depicting a female right hand grasping an object placed on a white table in front of a white wall (Figure 1A). All videos started with the hand resting on the table (500 ms) followed by the 3-s grasping action. In the conditions including AO without object, the videos had the same setup, however, no object was on the table and the hand mimicked grasping the object (Figure 1B). To recreate the kinematics of the object-grasping in the most accurate way, the actress had been trained to grasp the object for 10 times before filming the objectless action, and the filming occurred while the object was still visible (only for the actress) on the side of the table. In the encoding condition containing only the object, a photo of the object was presented in the same position but without the hand (Figure 1C). See https://osf.io/qy4jf/ for all materials.

*Motor Imagery Questionnaire-3* (*MIQ-3;* Williams et al., 2012). The MIQ-3 is a 12-item questionnaire to assess individuals’ ability to image four movements using internal visual imagery, external visual imagery and kinesthetic imagery. The MIQ-3 has been shown to have good psychometric properties, internal reliability and predictive validity. For the purposes of the present study, two out of the four scales were used, namely, internal visual imagery (first-person perspective; VI) and kinesthetic imagery (KI). Participants were instructed to perform each of the four movements (knee lift, jump, arm movement and waist bend, respectively), turn to the starting position and subsequently, perform the same action mentally (visually or kinesthetically, respectively). Finally, they were asked to rate the ease/difficulty with which they were able to do the imagery task on a Likert-scale ranging from 1 = very difficult to see/feel to 7 = very easy to see/feel. Visual imagery ability scores and kinesthetic imagery scores were obtained by computing the mean rating across all four performed actions, respectively. Internal consistency of the MIQ-3 was acceptable-good (total scale: *α* = 0.86; VI: *α* = 0.79; KI: *α* = 0.77).

### 2.4 Procedure

**Figure 2:**
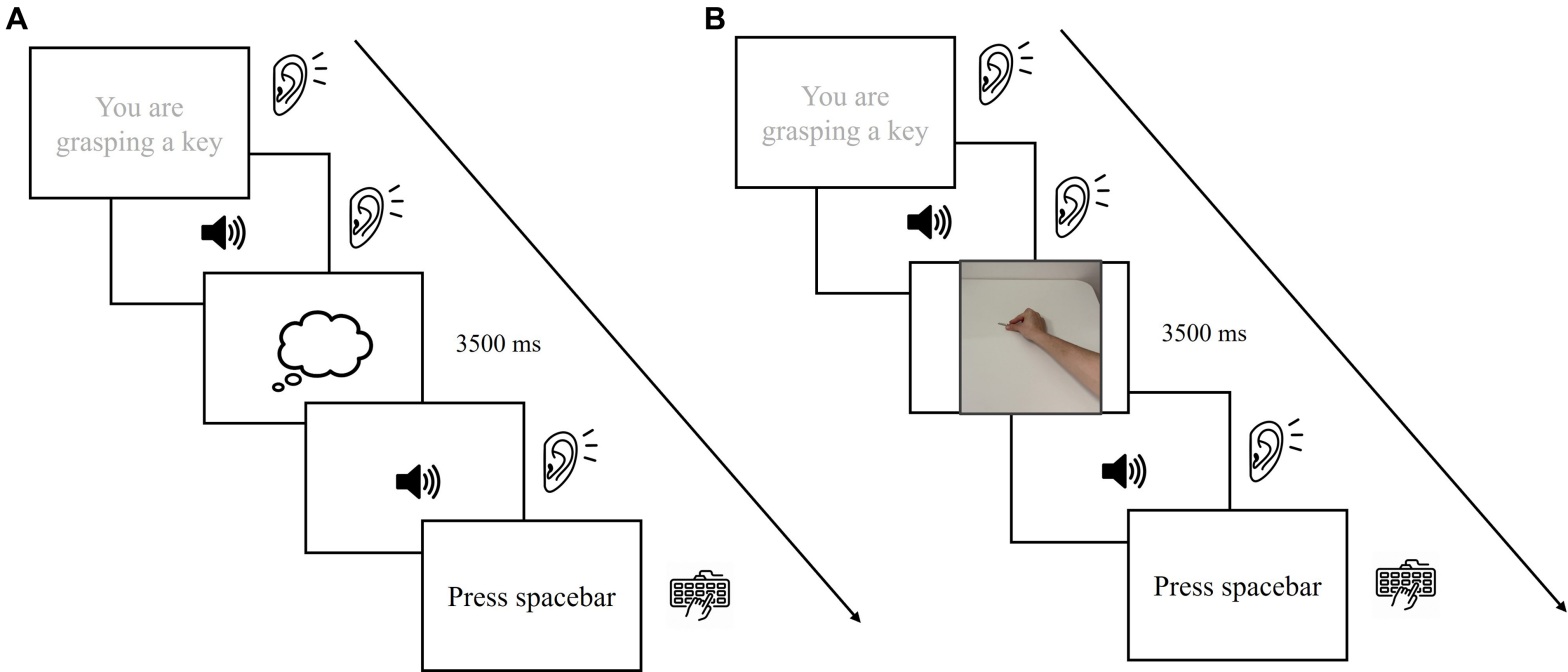
**(A)** Example trial of encoding conditions containing MI components. **(B)** Example trial of encoding conditions containing AO components.

After signing the informed consent and providing socio-demographic information, participants were presented with the instructions regarding the encoding part of the task (see the specific instructions for each condition in Supplementary material). The experiment presentation was built on E-Prime 3.0 and the whole procedure was carried out by participants on E-prime go (Version 1.0.1.44). Each encoding trial started with the oral presentation of one of the grasping-sentences. Subsequently, participants heard a “peep” sound which indicated the start of the second part of each trial and the sound was repeated after 3.5 s, indicating the end of the trial. As illustrated in Figure 2, the second part of the encoding trial differed between the various conditions. In all conditions with an AO component, a video depicting the grasping action was presented (see Materials) whilst in all conditions with a MI component, participants received the instruction to imagine performing the action in their mind in first person perspective and with the instruction to feel the associated bodily and motor sensations. In the mental rehearsal group, participants were instructed “after the beep, mentally repeat in your mind the sentence you just heard.”

After the second “beep” sound, participants were instructed to press the keyboard to proceed to the next trial. After two practice trials, participants performed the same trial procedure for all 13 grasping sentences. Subsequently, they watched a documentary on space, lasting 15 min and in which no human or biological movement were shown. Then, participants were instructed to say out loud all actions with objects they remembered from the first phase of the experiment (free recall); the order was not relevant. They had 90 s to orally list all actions on the objects (oral responses were chosen to avoid interfering with potential sensorimotor simulation linked to memory retrieval as it could be the case with manual keyboard/mouse responses, see, e.g., Ianì et al., 2023; Stockner et al., 2025a). Finally, they performed two scales of the MIQ-3.

## 3 Results

Recall responses were coded as 1 for each correctly retrieved object. One was also assigned to words that were conceptually the same (Cutica et al., 2014), but participants used a different term if the word was included in the Italian dictionary as a synonym (e.g., jug instead of carafe or jumper instead of sweater; 12.84% of the overall retrieved trials). Zero was assigned to all objects that were not retrieved by participants. Intrusions were excluded from the analysis.

A mixed effects model approach was adopted, and a Generalized Linear Mixed Model (GLMM) was run. Our model included AO, MI and OBJ as our fixed effects together with the two MIQ-scales (Kinesthetic Imagery KI and Visual Imagery VI), and sensorimotor characteristics of the objects (touch and visual scores) as covariates. Regarding random intercepts, the maximum model was initially applied (e.g., Barr, 2013) and reduced step-by-step to obtain convergence (the final model included random intercepts for both participants and stimuli with no random slopes). Finally, the resulting model was as follows: Recall ~ AO*MI*OBJ*(KI + VI) + (touchscores+visualscores) + (1 | Participant) + (1|Stimulus), implemented with the glmer() function from the *lme4* package (Bates et al., 2015) and tested with the Anova() function. *Post-hoc* comparisons were carried out with the emmeans function and the emtrends function (in case of continuous predictors) from the *emmeans* package (Russell, 2018). All statistical analyses were carried out using RStudio (RStudio Team, 2015, version 2024.04.1).

**Figure 3:**
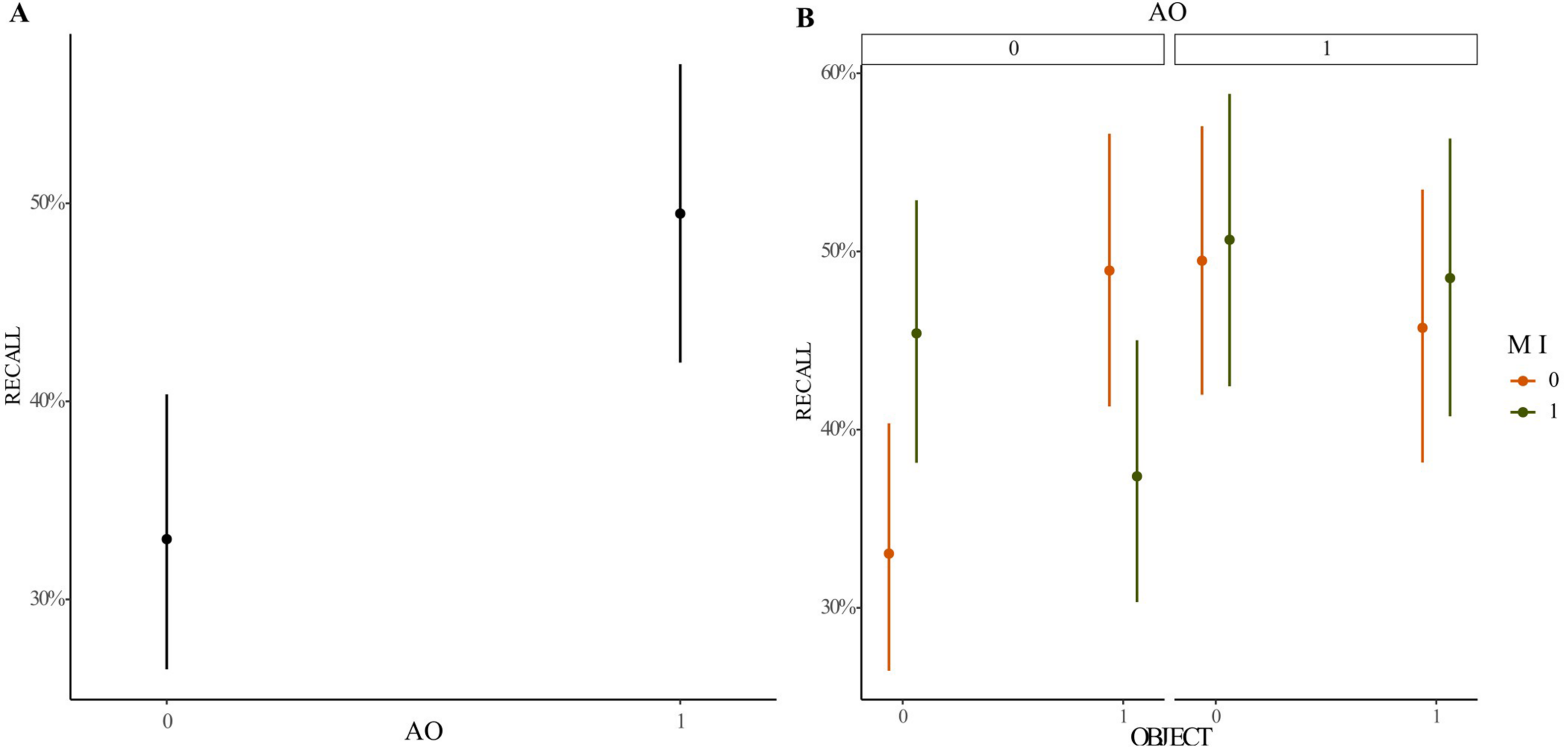
Predicted recall performance based on **(A)** action observation and **(B)** the interaction between action observation (AO), visual object presence (OBJ) and MI (motor imagery).

The GLMM on recall revealed a significant main effect of AO (*χ^2^*(1) = 7.76, *p* = 0.005; *OR* = 2.04), showing overall higher memory performance with the presence of AO compared to the absence of AO (see Figure 3A).

Moreover, a three-way interaction between AO, MI and OBJ was found (*χ^2^*(1) = 7.98, *p* = 0.0047; *OR* = 2.13) (see Figure 3B). More specifically, *post-hoc* comparisons revealed significant better memory performance for AO (vs. no AO) when MI and OBJ were either both absent or both present (*b* = −0.69, *SE* = 0.20, *z_ratio_* = −3.49, *p* = 0.0005, *OR* = 0.50 and *b* = −0.46, *SE* = 0.20, *z_ratio_* = −2.26, *p* = 0.024, *OR* = 0.63, respectively). On the other hand, the effect of AO (vs. no AO) was not significant when OBJ was present but MI absent (*p* = 0.52) nor with MI present and OBJ absent (*p* = 0.38). Moreover, MI (vs. no MI) improved memory performance significantly only when both AO and OBJ were absent (*b* = −0.52, *SE* = 0.20, *z_ratio_* = −2.67, *p* = 0.008, *OR* = 0.60). On the other hand, MI (vs. no MI) led to lower recall when participants could also see the object at encoding (OBJ present) but did not see the action (AO absent), (*b* = 0.47, *SE* = 0.20, *z_ratio_* = 2.36, *p* = 0.018, *OR* = 1.60). Finally, seeing the Object at encoding (vs. no OBJ) led to higher memory performance (*b* = −0.66, *SE* = 0.20, *z_ratio_* = −3.32, *p* = 0.0009, *OR* = 0.52) when both MI and AO were absent. No other comparisons were significant (all *p*s > 0.09), see Supplementary material for detailed statistics of null results. This three-way interaction and *post-hoc* comparisons confirm the crucial role of Action Observation, whilst also showing that each encoding condition, alone, similarly increases recall, and finally suggests that, when AO is absent, OBJ and MI can interfere with each other.

Although an overall two-way interaction was found between MI and OBJ (*χ^2^*(1) = 5.42, *p* = 0.0199, *OR* = 2.29), *post-hoc* comparisons, averaged over AO, did not show significant differences between the two different levels (all *p*s > 0.059).

**Figure 4:**
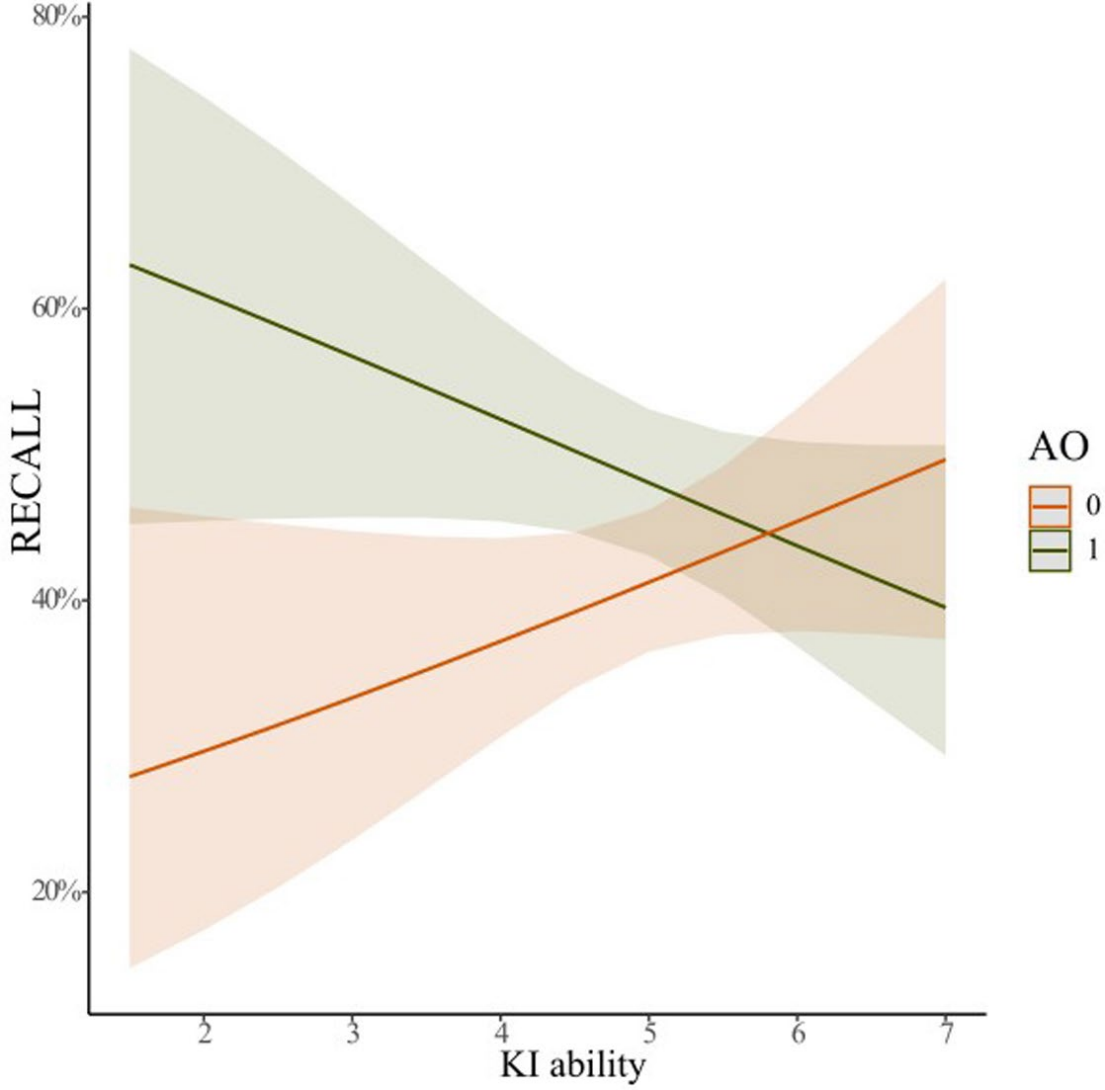
Interaction between KI (kinesthetic ability scale, MIQ-3) and AO (action observation) in predicting recall performance.

Further, individual characteristics in the ability to create motor images (i.e., kinesthetic imagery ability) interacted with AO in predicting recall (*χ^2^*(1) = 4.99, *p* = 0.025, *OR* = 0.78). *Post-hoc* analysis shows that the slopes between conditions with AO and conditions without AO were significantly different (*b* = 0.35, *SE* = 0.16, *z_ratio_* = 2.23, *p* = 0.026, *OR* = 1.41). As illustrated in Figure 4, memory performance appears to decrease as KI ability increases in conditions with AO, suggesting an association between a high level of KI and low encoding in AO. No other main effects or interaction effects were found (all *p*s > 0.07).

## 4 Discussion

In the present study we investigated the role of different sensorimotor simulations in a free recall task. By applying a between-subject design, in which we manipulated action observation (AO), motor imagery (MI) and visual object presence (OBJ), we provided evidence of the superior role of AO in free recall of objects. When AO was present at encoding, neither motor imagery (MI) nor the additional presence of the object (OBJ) had any effect. This result is somewhat in line with findings of Senkfor et al. (2002) who found AO, after enactment, to be most beneficial for source memory and other fields of cognition, such as motor learning, in which AO has shown to be most beneficial (e.g., Gatti et al., 2013). While MI and OBJ each improved object memory when presented alone at encoding, their effects only emerged in the absence of a concurrent observed action. AO, instead, improved memory both in the absence of both object and MI (confirming its beneficial role compared to non-sensorimotor conditions, e.g., Ianì and Bucciarelli, 2017; Steffens, 2007), but also in the presence of both object and MI. The fact that AO improved memory also when the other two encoding conditions were present (AO + MI + OBJ) can be interpreted in several ways (we remind that there was no ceiling effect). According to the most parsimonious explanation, when participants observe the mere action, all necessary information is already in it, and a concomitant act of imagination of the action, or observation of the object does not add any further useful information. On the other hand, AO seems to provide additional useful information, even when the other two (MI and OBJ) are already present. This is in line with literature in other areas, such as motor learning or action execution, in which the concurrent sensorimotor simulation AO + MI (including the object) elicited the strongest effects (Eaves et al., 2024).

However, in our experiment, the presence of AO did not improve memory in all conditions. No additional improvement was obtained by adding AO to either MI *or* OBJ, when the other was absent, respectively. Although speculative, also considering our power-analysis was not specifically designed to detect a three-way-interaction, it is possible to hypothesise that the lack of additional improvement in the first case (AO does not improve memory when MI is present and OBJ absent) can be ascribed to a form of conflicting AOMI. To clarify, Vogt et al. (2013) presented a dual-simulation theory explaining how two sensorimotor processes (AO and MI) can be represented simultaneously in the brain, but also highlight the possibility of a competition between the two processes, especially in the case in which the observed and imaged action are not exactly the same (conflicting AOMI). Thus, adding AO to MI seems to be advantageous only when the object is present, potentially leading to an increased overlap between the imagined and observed action. Conversely, the risk for creating conflicting AOMI may have been increased in AO + MI when no object was present. Although speculative, we hypothesize that the object might have served as an “anchor” in guiding imagery whilst observing the action. It should be noted that we did not directly test for a potential AOMI conflict; consequently, we lack empirical evidence on this issue, and the hypothesis advanced above remains speculative. Further research would be needed to better understand the underlying beneficial effects of AOMI when the object is included (vs. no object). Moreover, adding AO did not improve memory when the visual object was present and MI was absent, either. This result could be potentially explained by our experimental setup in which the hand in AO conditions directly interacted with the object only in the very last part of the video whilst they saw the object presented on the screen for the whole time. Thus, we cannot exclude that also in the AO condition participants focused (mainly) their attention on the object and not on the action, in which case AO could not provide any additional contribution to memory performance.

Finally, even though kinesthetic ability was not experimentally manipulated, the interaction between AO and the individual kinesthetic imagery ability provides an additional insight on the relationship between different types of simulations. More specifically, results showed that higher kinesthetic ability corresponds to low memory performance in the AO condition. This could be interpreted in the light of internally directed attention, which has been linked to mental imagery (Cooper et al., 2003) and which could have hindered the attention to the external stimulus, involved in action observation. To our knowledge, there is no literature in this regard yet. However, Bonnet et al. (2022) results, obtained in a language comprehension task, can be interpreted in line with our findings and explanation. The authors found that a kinesthetic motor-imagery training (thus, increasing kinesthetic motor imagery ability) interfered with the processing of action words, rather than facilitating it. Regarding our specific result on AO, it is also worth noting that research has hypothesized that AO may also automatically elicit a form of spontaneous or ‘involuntary’ mental simulation or motor resonance (Romano-Smith et al., 2018) which could differ in terms of nature, temporality and sensorimotor features from the simulation involved in voluntary mental imagery. Interestingly, it is also worth noting that we did not find a significant (interaction) effect with visual imagery ability, confirming the multidimensionality of mental imagery ability (e.g., Blazhenkova, 2016). Unlike other forms of mental simulation, mental imagery appears to require a multistep neurocognitive process involving different neural systems, such as visual and motor areas as well as prefrontal circuits. This results in highly heterogeneous mental representations that can vary from each other across several dimensions (for a discussion, see Stockner et al., 2025b). Thus, it is reasonable that KI ability differs from VI ability, and interacts differently with simulation processes involved in action observation. Future research in this regard is needed to investigate the mechanisms involved in the interaction, and thus, the interplay between different types of sensorimotor simulations.

### 4.1 Limitations

When interpreting our results, it should be noted that our study is not without limitations. First, it is important to highlight that our results refer to a specific type of stimuli (i.e., grasping sentences) and we cannot exclude that the encountered AO effects may be related to this type of stimuli. In other words, future research would be needed to verify whether our results also extend to other types of stimuli (different types of actions). Second, whilst our free recall task allowed us to have a pure measure of memory retrieval, it would be highly informative to extend our research with a recognition task, providing insight into different memory processes such as reaction times or metacognition which have been shown to be especially useful in research on embodied memory (Convertino et al., 2025; Limata et al., 2023). Finally, the present study lacks a direct manipulation check to confirm that participants actually engaged in motor imagery as instructed. Although the MIQ-3 provides a measure of imagery ability, it does not verify whether participants complied with the task during the encoding phase. Moreover, kinesthetic imagery ability was measured with the MIQ-3 rather than experimentally manipulated; future studies should manipulate this dimension to support causal interpretations of the relationship between kinesthetic imagery and the effectiveness of AO.

To summarize, our results overall highlight the predominant role of AO as well as the dynamic interplay between motor imagery and visual object presence components. Further, we provide the first evidence that kinestethic imagery ability can impair memory performance based on action observation: the better participants are able to create motor images, the less they benefit from action observation, i.e., only participants with lower KI abilities benefit from AO.

## Data Availability

The datasets presented in this study can be found in online repositories. The names of the repository/repositories and accession number(s) can be found at: https://osf.io/qy4jf/.

## Appendix

**Table A1:** Original stimuli (*with English translations*) of the two experimental blocks and associated grasping categories, matched between the two experimental blocks.

Object	Experimental block	Grasping category
ago (*needle*)	1	1
bicchiere (*cup*)	1	2
libro (*book*)	1	3
matita (*pencil*)	1	4
hamburger (*hamburger*)	1	5
chiave (*key*)	1	6
forbici (*scissors*)	1	7
vaso (*vase*)	1	8
bollitore (*kettle*)	1	9
padella (*frying-pan*)	1	10
lettera (*letter*)	1	11
scolapasta (*colander*)	1	12
cuscino (*pillow*)	1	13
forcina (*hair pin*)	2	1
muffin (*muffin*)	2	2
quaderno (*notebook*)	2	3
penna (*pen*)	2	4
posacenere (*ashtray*)	2	5
moneta (*coin*)	2	6
spillatrice (*stapler*)	2	7
bottiglia (*bottle*)	2	8
caraffa (*jug*)	2	9
asciugacapelli (*hair drye*r)	2	10
carta (*paper*)	2	11
pentola (*pan*)	2	12
maglia (*sweater*)	2	13
Lanterna (*lantern*)	1	Practice trial
Ombrello (*umbrella*)	2	Practice trial
