# Supplementary material for: Embodied simulations improve memory, but which is the most effective? The crucial role of action observation and its dynamic interplay with motor imagery ability
Source: Front Psychol. 2026 Jul 22;17:1889010. doi: 10.3389/fpsyg.2026.1889010 (PMC13438388; doi:10.3389/fpsyg.2026.1889010)
Supplement: Supplementary file 1 [file Data_Sheet_1.PDF]

Supplementary Materials

1. Encoding instructions of the eight experimental groups (translated from Italian to English)

All encoding instructions started with "Thank you for participating in this experiment on how we understand actions associated with objects. In this first phase, you will hear a series of sentences that describe actions" and ended with "We ask you to perform this task carefully because you will then be asked questions about the actions".

The specific encoding instruction for each experimental group was as follows:

AO - no MI - no OBJ

After each sentence, you will hear a "beep" that announces the beginning of a video in which you will see the action depicted in front of you. At the end of the video, you will hear a second beep".

AO - MI - no OBJ

After each sentence, you will hear a "beep" that announces the beginning of a video in which you will see the corresponding action depicted in front of you. At the same time, while you watch the video, we ask you to imagine yourself performing the action described in the sentence and depicted in the video. Imagine performing the same action that you are observing from your perspective, as if you were looking through your eyes and seeing your hands performing the action in front of you. We ask you to also imagine the bodily sensations that you would feel while performing the action. You will hear a second "beep" that will indicate the end of the video and the imagination task.

AO - no MI - OBJ

After each sentence, you will hear a "beep" that announces the beginning of a video in which you will see the action with the object depicted in front of you. At the end of the video, you will hear a second beep.

AO - MI - OBJ

After each sentence, you will hear a "beep" that announces the beginning of a video in which you will see the corresponding action depicted in front of you. At the same time, while you watch the video, we ask you to imagine yourself performing the action described in the sentence and depicted in the video. Imagine performing the same action that you are observing from your perspective, as if you were looking through your eyes and seeing your hands performing the action in front of you. We ask you to also imagine the bodily sensations that you would feel while performing the action with the object. You will hear a second "beep" that will indicate the end of the video and the imagination task.

no AO - MI - no OBJ

After each sentence, we will ask you to imagine performing the action of the sentence you heard. Specifically, as soon as you hear a "beep", we ask you to imagine that the object of the sentence appears on the table in front of you and that you perform the action that was indicated in the sentence. Imagine performing this action from your perspective, as if you were looking through your eyes and seeing your hands performing the action in front of you. We ask you to also imagine the bodily sensations that you would feel while you performed the action with the object. You will hear a second "beep" that will indicate the end of the task.

no AO - MI - OBJ

After each sentence, you will hear a "beep" after which you will see an image of the corresponding object in front of you. At the same time, while you are looking at the image, we ask you to imagine performing the action that was indicated in the sentence. Imagine performing this action from your perspective, as if you were looking through your eyes and seeing your hands performing the action in front of you. We ask you to also imagine the bodily sensations that you would feel while you performed the action with the object. You will hear a second "beep" that will indicate the end of the task.

no AO - no MI - OBJ

After each sentence, you will hear a "beep" after which you will see an image of the corresponding object in front of you that we ask you to observe. You will hear a second "beep" which will indicate the disappearance of the image.

no AO - no MI -no OBJ (mental rehearsal)

After each sentence, you will hear a “beep” after which we ask you to repeat in your mind the sentence you heard. You will hear a second "beep" that will indicate the end of the task.

2. Descriptive statistics MIQ-3

Scale	Mean	SD	Median	Minimum	Maximum
Kinesthetic Imagery	4.92	1.14	5.00	1.5	7.00
Visual imagery	4.98	1.20	5.25	1.75	7.00

3. Detailed post hoc comparisons

Three-way-interaction AO x MI x OBJ (Tables 1-3):

Table 1. *Contrasts of AO presence (AO = 1 vs. AO = O) based on MI and OBJECT*

MI	OBJECT	Contrast	Estimate	SE	df	z.ratio	p.value
0	0	AO0 - AO1	-0.686	0.197	Inf	-3.488	0.0005
1	0	AO0 - AO1	-0.172	0.198	Inf	-0.869	0.3849
0	1	AO0 - AO1	0.126	0.197	Inf	0.639	0.5231
1	1	AO0 - AO1	-0.457	0.202	Inf	-2.260	0.0238

Table 2. *Contrasts of MI presence (MI = 1 vs. MI = O) based on AO and OBJECT*

AO	OBJECT	Contrast	Estimate	SE	df	z.ratio	p.value
0	0	MI0 - MI1	-0.52058	0.195	Inf	-2.668	0.0076
1	0	MI0 - MI1	-0.00634	0.200	Inf	-0.032	0.9747
0	1	MI0 - MI1	0.47071	0.200	Inf	2.359	0.0183
1	1	MI0 - MI1	-0.11155	0.199	Inf	-0.560	0.5758

Table 3. *Contrasts of OBJECT presence (OBJECT = 1 vs. OBJECT = O) based on AO and MI*

MI	AO	Contrast	Estimate	SE	df	z.ratio	p.value
0	0	OBJECT0 - OBJECT1	-0.6604	0.199	Inf	-3.318	0.0009
1	0	OBJECT0 - OBJECT1	0.3308	0.195	Inf	1.695	0.0900
0	1	OBJECT0 - OBJECT1	0.1516	0.195	Inf	0.778	0.4364
1	1	OBJECT0 - OBJECT1	0.0464	0.205	Inf	0.227	0.8207

Two way interaction: MI x OBJECT (Tables 4-5):

Table 4. *Contrasts of OBJECT presence (OBJECT = 1 vs. OBJECT = O) based on MI*

MI	Contrast	Estimate	SE	df	z.ratio	p.value
0	OBJECT0 - OBJECT1	-0.254	0.139	Inf	-1.826	0.0679
1	OBJECT0 - OBJECT1	0.189	0.141	Inf	1.334	0.1823

Table 5. *Contrasts of MI presence (MI = 1 vs. MI= O) based on OBJECT*

OBJECT	Contrast	Estimate	SE	df	z.ratio	p.value
0	MI0 - MI1	-0.263	0.140	Inf	-1.885	0.0595
1	MI0 - MI1	0.180	0.141	Inf	1.274	0.2028
